# Supplementary material for: Content-rich biological network constructed by mining PubMed abstracts
Source: BMC Bioinformatics. 2004 Oct 8;5:147. doi: 10.1186/1471-2105-5-147 (PMC528731; doi:10.1186/1471-2105-5-147)
Supplement: Additional File 2 — The original results of the above study (non-essential files are deleted to keep the file size under the limit set by BMC bioinformatics). [file 1471-2105-5-147-S2.bz2 › chilibotAdditionalFile2/dip20/78ID7565724E290/html/WAS_NCK1.html]

 


 **WAS** and **NCK1** 
  
Found 20 abstracts in PubMed, retrieved 20.  
 

 What does Google say? 
 PDF only 
| .edu only 

---

**Interactive relationship** (e.g. stimulation, inhibition, etc)

**Neutral relationship**- Both phosphorylation of WASp  [ **WAS** ]  and its interaction with Grb2, as well as with another adapter protein Nck  [ **NCK1** ] , remain constitutive in serum starved or epidermal growth factor stimulated cells.  Ref: 9307968 Mol Biol Cell, 1997
- We also show that Nck  [ **NCK1** ]  and N WASP  [ **WAS** ]  are essential for the actin based motility of vaccinia virus.  Ref: 10553910 Nature, 1999
- and another through Nck  [ **NCK1** ]  that promotes the recruitment of WASP  [ **WAS** ] .  Ref: 11739662 J Cell Sci, 2001
- Upon induction of phagocytosis, a large molecular complex, consisting in part of Ena VASP proteins, the Fyn binding SLP 76 associated protein Fyb SLAP, Src homology 2 SH2 domain containing leukocyte protein of 76 kDa SLP 76, Nck  [ **NCK1** ] , and the Wiskott Aldrich syndrome protein  [ **WAS** ]  WASP  [ **WAS** ] ,  **was**  formed.  Ref: 11739662 J Cell Sci, 2001
- Phosphatidylinositol biphosphate PIP2 induced vesicle movement depends on N WASP  [ **WAS** ]  and involves Nck  [ **NCK1** ] , WIP, and Grb2.  Ref: 12147689 J Biol Chem, 2002
- The recruitment and activation of WASP  [ **WAS** ]  are coordinated by tyrosine phosphorylated Src homology 2 domain containing leukocyte protein of 76 kDa, which functions as a scaffold, bringing Nck  [ **NCK1** ]  and WASP  [ **WAS** ]  into proximity with Vav 1 and Cdc42 GTP.  Ref: 12874226 J ImmunolJ Immunol, 2002
- Evidence for a molecular complex consisting of Fyb SLAP, SLP 76, Nck  [ **NCK1** ] , VASP and WASP  [ **WAS** ]  that links the actin cytoskeleton to Fcgamma receptor signalling during phagocytosis.  Ref: 11739662 J Cell Sci, 2001
- This process resulting in the motility of intracellular vesicles at the tips of actin comets involved the recruitment of the Src homology 3 SH3 SH2 adaptor proteins Nck  [ **NCK1** ]  and Grb2 as well as of WASP  [ **WAS** ]  interacting protein WIP.  Ref: 12147689 J Biol Chem, 2002
- Tyrosine phosphorylation of A36R results in a direct interaction with the adaptor protein Nck  [ **NCK1** ]  and the recruitment of the Ena VASP family member N WASP  [ **WAS** ]  to the site of actin assembly.  Ref: 10553910 Nature, 1999
- This observation, and the results presented here, suggest that  **WAS**  is the result of defects in signal transduction pathways regulated by Cdc42 Rac and Nck  [ **NCK1** ] .  Ref: 8805223 Curr Biol, 1996

**Non-interactive relationship** (e.g. studied together, co-existance, homology, etc.)

- Nck  [ **NCK1** ]  binds through its second SH3 domain to WIP, and Nck  [ **NCK1** ]  binds to WIP at a site amino acids 321 415 that differs from the WASP  [ **WAS** ]  binding site amino acids 416 488.  Ref: 9694849 J Biol Chem, 1998
- WASP  [ **WAS** ]  has recently been found to bind to the Src homology 3 SH3 domain of the adapter protein Nck  [ **NCK1** ] .  Ref: 8805223 Curr Biol, 1996
- The Nck  [ **NCK1** ]  Src homology SH 2 3 adaptor binds via its SH3 domains to a proline rich region on WASP  [ **WAS** ]  and N WASP  [ **WAS** ]  and has been implicated in recruitment of these proteins to sites of tyrosine phosphorylation.  Ref: 11340081 J Biol Chem, 2001
- Moreover, activated Nck  [ **NCK1** ]  interacted with Cas and WASP  [ **WAS** ]  as determined by co immunoprecipitation, and FAK also bound to Cas.  Ref: 12135674 Exp Hematol, 2002
- We show here that Nck  [ **NCK1** ]  SH3 domains dramatically stimulate the rate of nucleation of actin filaments by purified N WASP  [ **WAS** ]  in the presence of Arp2 3 in vitro.  Ref: 11340081 J Biol Chem, 2001
- In activated T cells, Fyb SLAP associates with Ena VASP family proteins and is present within biochemical complexes containing WASP  [ **WAS** ] , Nck  [ **NCK1** ] , and SLP 76.  Ref: 10747096 J Cell Biol, 2000
- Nck  [ **NCK1** ]  and phosphatidylinositol bisphosphate synergistically activate actin polymerization through the N WASP  [ **WAS** ]  Arp2 3 pathway.  Ref: 11340081 J Biol Chem, 2001
- The Wiskott Aldrich syndrome protein  [ **WAS** ]  interacting protein WIP binds to the adaptor protein Nck  [ **NCK1** ] .  Ref: 9694849 J Biol Chem, 1998
- Wiskott Aldrich syndrome protein  [ **WAS** ]  physically associates with Nck  [ **NCK1** ]  through Src homology 3 domains.  Ref: 7565724 Mol Cell Biol, 1995
- Nck  [ **NCK1** ]  stimulated actin nucleation by N WASP  [ **WAS** ] .Arp2 3 complexes is further stimulated by phosphatidylinositol bisphosphate, but not by GTP Cdc42, suggesting that Nck  [ **NCK1** ]  and Cdc42 activate N WASP  [ **WAS** ]  by redundant mechanisms.  Ref: 11340081 J Biol Chem, 2001
- Our previous studies have shown that N WASP  [ **WAS** ]  is required for the actin based motility of vaccinia virus and is recruited via Nck  [ **NCK1** ]  and WIP.  Ref: 12007418 Curr Biol, 2002
- Nck  [ **NCK1** ]  is required for the recruitment of both neural Wiskott Aldrich syndrome protein  [ **WAS** ]  N WASP  [ **WAS** ]  and the actin related protein Arp 2 3 complex to the EPEC pedestal, directly linking Tir to the cytoskeleton.  Ref: 11533668 Nat Cell Biol, 2001
- The Grb2 pathway alone is therefore unable to nucleate actin tails, as its recruitment requires the prior recruitment of N WASP  [ **WAS** ]  by Nck  [ **NCK1** ] .  Ref: 12007418 Curr Biol, 2002
- Here, we show that WASP  [ **WAS** ]  recruitment occurs through binding to the C terminal Src homology 3 domain of Nck  [ **NCK1** ] .  Ref: 12874226 J ImmunolJ Immunol, 2002
- Unlike WASP  [ **WAS** ] , which bound the SH3 domains of several signaling proteins, PRK2 specifically bound to the middle SH3 domain of NCK  [ **NCK1** ]  and weakly that of phospholipase Cgamma.  Ref: 8910519 J Biol Chem, 1996
- SLP 76 Coordinates Nck  [ **NCK1** ]  Dependent Wiskott Aldrich Syndrome Protein  [ **WAS** ]  Recruitment with Vav 1 Cdc42 Dependent Wiskott Aldrich Syndrome Protein  [ **WAS** ]  Activation at the T Cell APC Contact Site.  Ref: 12874226 J ImmunolJ Immunol, 1996
- In this report, we demonstrate that recombinant Nck  [ **NCK1** ]  precipitates endogenous WIP, a novel proline rich protein that interacts with the Wiskott Aldrich syndrome protein  [ **WAS** ]  WASP  [ **WAS** ] , from BJAB cell lysates.  Ref: 9694849 J Biol Chem, 1998
- The SH3 SH3 SH3 SH2 adapter protein Nck  [ **NCK1** ]  links receptor tyrosine kinases, such as EGF and PDGF receptors, to downstream signaling pathways, among which p21cdc42 rac activated kinase cascade, Sos activated Ras signaling and the human Wiskott Aldrich Syndrome protein  [ **WAS** ]  WASp  [ **WAS** ]  mediated actin cytoskeleton changes, have been implicated.  Ref: 9362449 Oncogene, 1997
- CONCLUSIONS These data suggest that WASP  [ **WAS** ] , Cas, Nck  [ **NCK1** ] , and FAK may play a role in SDF 1alpha induced migration of the T cell line, Jurkat.  Ref: 12135674 Exp Hematol, 2002
